# Epidemiology and Survival of Patients With Brainstem Gliomas: A Population-Based Study Using the SEER Database

**DOI:** 10.3389/fonc.2021.692097

**Published:** 2021-06-11

**Authors:** Huanbing Liu, Xiaowei Qin, Liyan Zhao, Gang Zhao, Yubo Wang

**Affiliations:** ^1^ Department of Neurosurgery, First Hospital of Jilin University, Changchun, China; ^2^ Department of Clinical Laboratory, Second Hospital of Jilin University, Changchun, China

**Keywords:** brainstem glioma, epidemiology, survival, SEER Program, CNS disease

## Abstract

**Background:**

Brainstem glioma is a primary glial tumor that arises from the midbrain, pons, and medulla. The objective of this study was to determine the population-based epidemiology, incidence, and outcomes of brainstem gliomas.

**Methods:**

The data pertaining to patients with brainstem gliomas diagnosed between 2004 and 2016 were extracted from the SEER database. Descriptive analyses were conducted to evaluate the distribution and tumor-related characteristics of patients with brainstem gliomas. The possible prognostic indicators were analyzed by Kaplan-Meier curves and a Cox proportional hazards model.

**Results:**

The age-adjusted incidence rate was 0.311 cases per 100,000 person-years between 2004 and 2016. A total of 3387 cases of brainstem gliomas were included in our study. Most of the patients were white and diagnosed at 5-9 years of age. The most common diagnosis confirmed by histological review was ependymoma/anaplastic ependymoma. The median survival time was 24 months. Patients with tumors less than 3 cm in size had a better prognosis. Surgery was effective at improving overall survival. There was no evidence that radiotherapy and chemotherapy improved overall survival.

**Conclusion:**

Brainstem gliomas can be diagnosed at any age. Ependymoma/anaplastic ependymoma is the most common pathological diagnosis. The prognosis is poor, and timely diagnosis and surgery are effective at improving the prognosis. We suggest that more attention should be given to the treatment of patients with brainstem gliomas.

## Introduction

Gliomas are primary brain tumors that are thought to arise from neuroglial stem or progenitor cells (1). Gliomas are the most common malignant primary brain tumor, and 4.3% of gliomas are localized at the brainstem (2). Brainstem glioma is a primary glial tumor that arises from the midbrain, pons, and medulla. In most instances, the term refers to a highly aggressive tumor of the pons (3). Diffuse intrinsic pontine glioma has been reported to account for ~75% of brain stem tumors in children (2). Because of poor survival, brainstem glioma has been a main research focus for decades (4).

The Surveillance, Epidemiology, and End Results (SEER) program of the National Cancer Institute represents approximately 35% of the US population (based on the 2000 census) (5). Our goal is to use SEER data to analyze the epidemiology and survival of patients with brainstem gliomas in the United States.

## Methods

### Data Extraction and Incidence Rates

The data from SEER are available to the public for research purposes. Therefore, ethics committee approval and informed consent were not necessary to perform the analyses. Patients with a diagnosis of primary brainstem gliomas were included. The term glioma was defined by setting the variable “Histology recode - broad groupings” as “9380-9489: gliomas”. Brainstem gliomas were defined by setting the variable “Primary Site - labeled” as “C71.7-Brainstem”. The research period was set from 2004 to 2016. Age-adjusted incidence rates (directly standardized to the 2000 US standard population) between 2004 and 2016 were retrieved from the SEER 18 database (November 2019 submission) (6). The detailed patient data were obtained from SEER 18 Regs Custom Data (November 2018 submission) (7). All the data were obtained by using the SEER*Stat 8.3.8 program.

### Variables and Population Analysis

The demographic and clinical features included age at diagnosis (0-19 years, ≥20 years), sex (Male, Female), race (White, Black, Asian or Pacific Islander, American Indian/Alaska Native, Unknown), Purchased/Referred Care Delivery Area (PRCDA) Region (Alaska, eastern region, north plains, Pacific coast, southwestern region), tumor size (≤3 cm, >3 cm and Unknown), diagnostic confirmation (positive microscopic confirm, others), behavior code (benign, borderline malignancy, malignant) according to the International Classification of Diseases for Oncology 3 (ICD-O-3), surgery (Yes, None/Unknown), radiation therapy (Yes, None/Unknown), chemotherapy (Yes, None/Unknown), survival months and vital status. The pathology types (according to the code of “Histology recode - Brain groupings”) of the patients diagnosed with positive microscopic confirmation were analyzed. Descriptive analyses were conducted to evaluate the distribution and tumor-related characteristics of patients with brainstem gliomas. Bar graphs and pie charts were also used to further describe the distribution of patients.

### Survival Analysis

The Kaplan-Meier method was used to estimate overall survival (OS) at 1, 3, 5, and 10 years. Survival time was defined as the time from diagnosis to death from any cause. We also used this method to estimate the OS in different groups. The differences between the curves were analyzed by the log‐rank test. Univariate and multivariate Cox proportional hazard models were performed to estimate the hazard ratios (HRs) and 95% confidence intervals (CIs) to analyze the independent prognostic factors associated with OS in patients with brainstem gliomas, and statistical significance was defined as p < 0.05. All the data were analyzed by IBM SPSS Statistics 25 software (IBM Corporation, Armonk, New York, USA).

## Results

### Population Analysis

**Figure 1:**
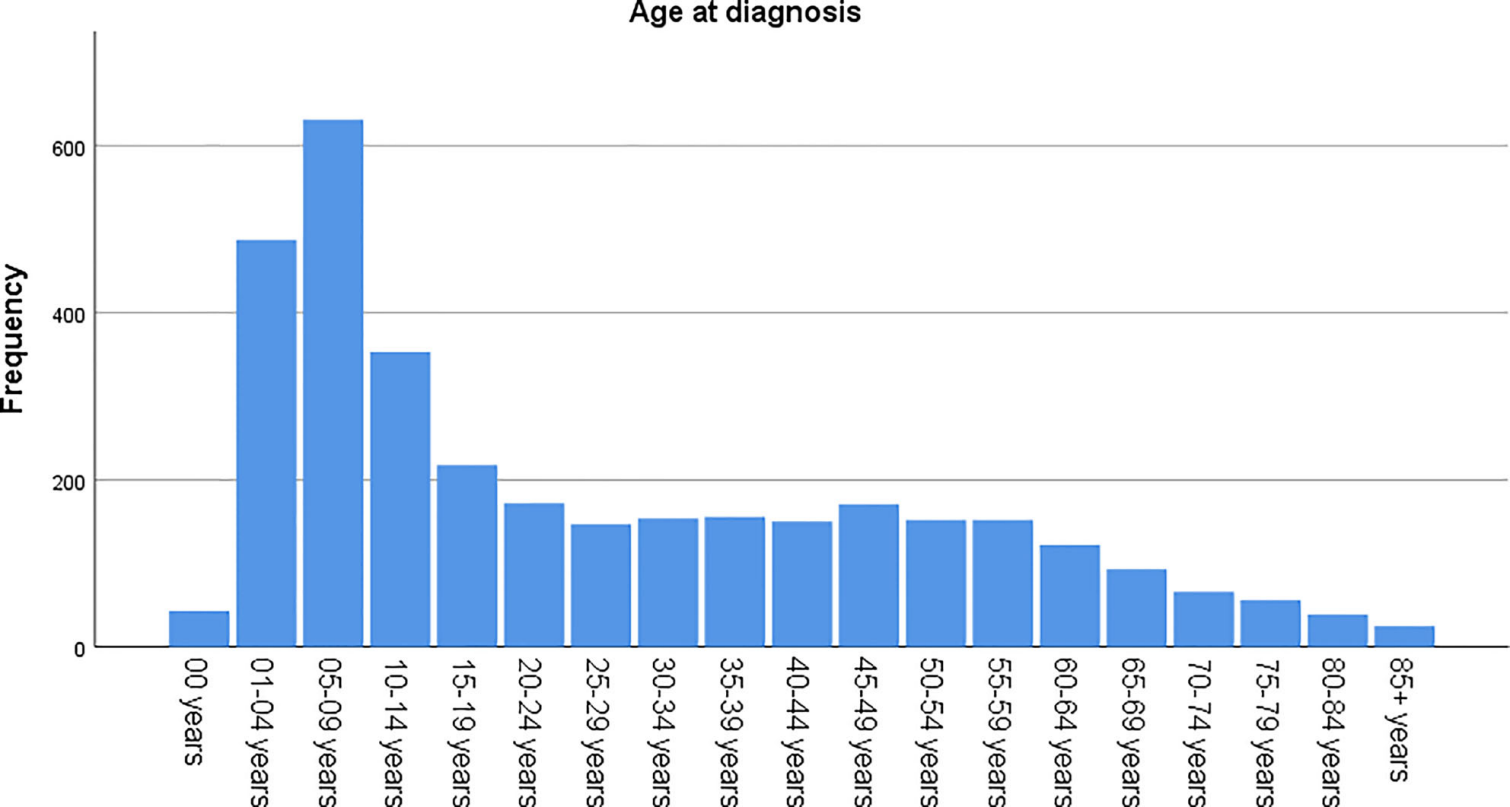
Age distribution of the patients at diagnosis.

**Figure 2:**
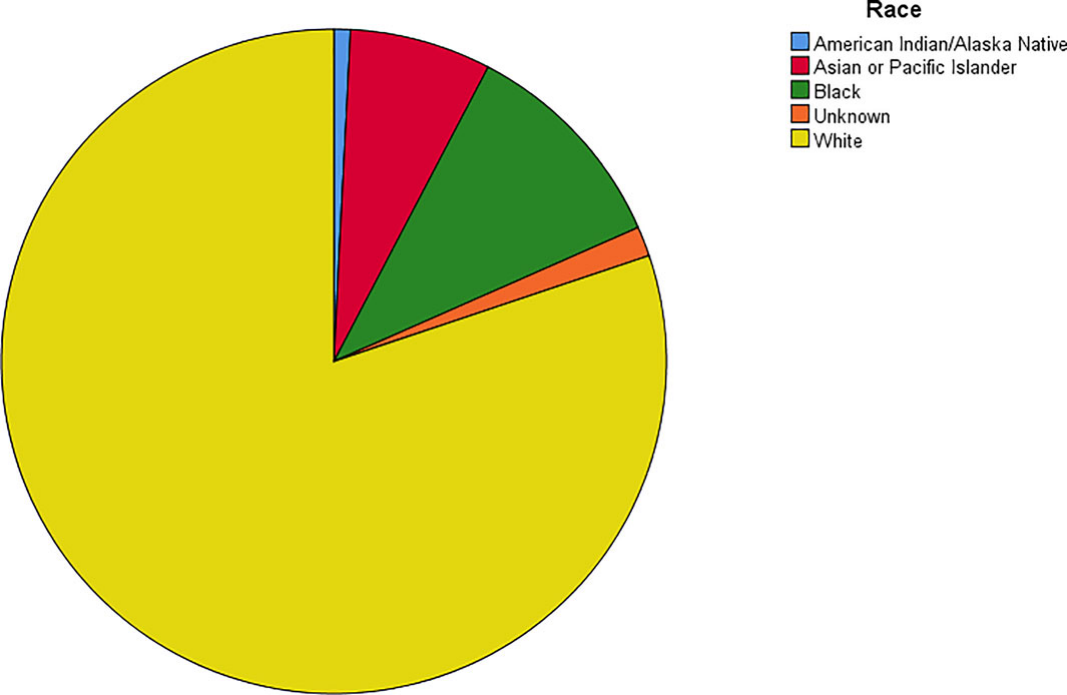
Racial distribution of the patients.

**Figure 3:**
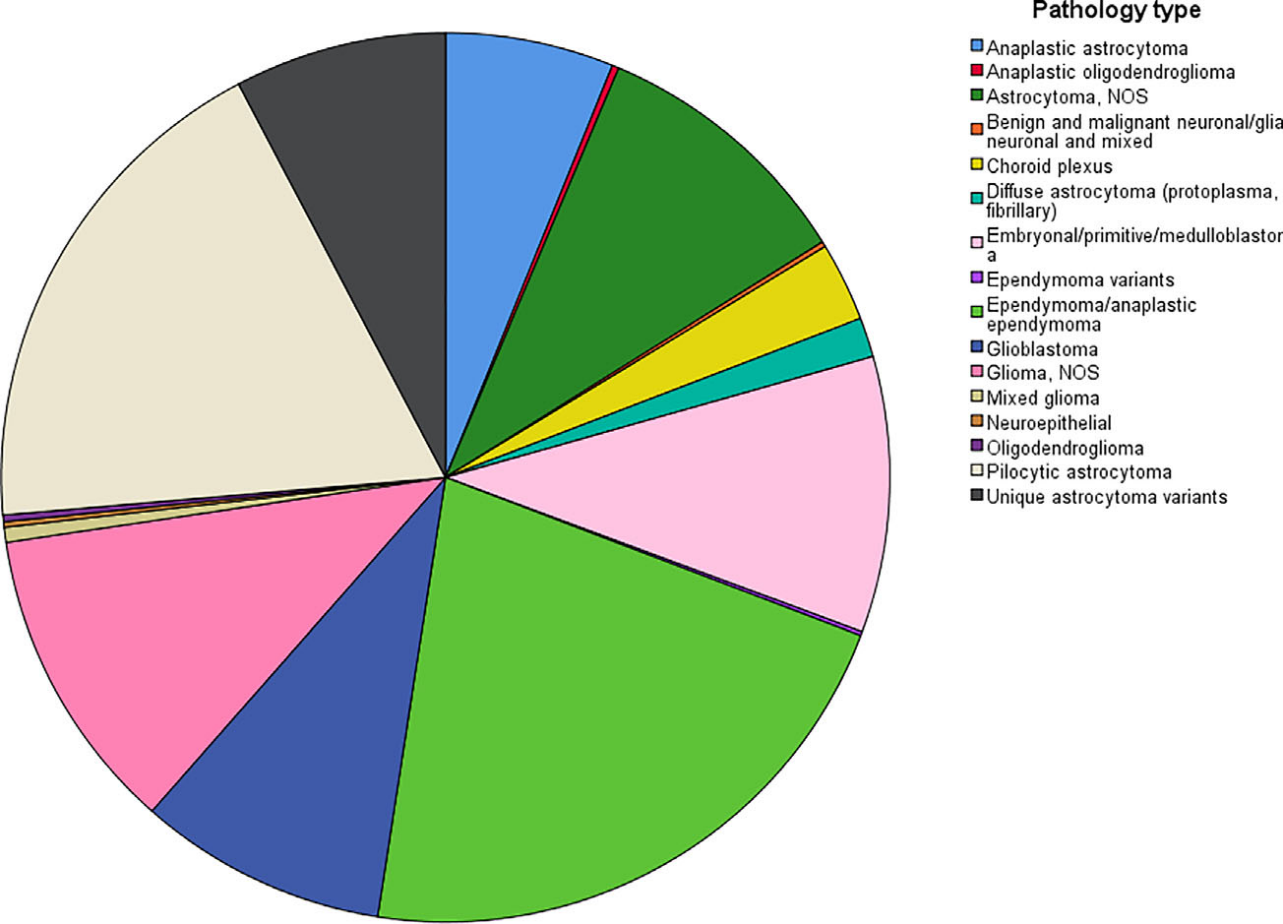
The distribution of the patients with different pathological types.

The age-adjusted incidence rate was 0.311 cases per 100,000 person-years between 2004 and 2016. A total of 3387 cases of brainstem gliomas were indexed between 2004 and 2016. The demographic and clinical characteristics of the patients are shown in **Table 1**. There were 1535 female patients (45.3%) and 1852 male patients (54.7%). The median age was 18 years (range 0 to 103 years). The majority of patients were diagnosed when they were between 5 and 9 years old, and the distribution of patient age at diagnosis is shown in a histogram (**Figure 1**). Children and adolescents (0-19 years old) accounted for 34.3% of all patients. White patients accounted for 80.2% of all patients (**Figure 2**). Most of the patients were from the Pacific coast (n=1740, 51.4%) and eastern region (n=1187, 35.0%). According to ICD-0-3, most of the tumors were malignant (n=3040, 89.8%). Among this cohort, 2023 cases were diagnosed with positive microscopic confirmation. We analyzed pathology type among these patients, and the results are shown in a pie chart (**Figure 3**). We found that ependymoma/anaplastic ependymoma (n=438, 21.7%) and pilocytic astrocytoma (n=377, 18.6%) were the most common pathology types. A total of 37.2% of the tumors were less than 3 cm in size, and 33.5% of the tumors were larger than 3 cm. Radiation was the first choice of therapy for patients with brainstem gliomas. Surgery was performed in 1479 (43.7%) cases, radiation therapy was performed in 1746 (51.6%) cases, and chemotherapy was performed in 1166 (34.4%) cases. The median survival time was 24 months (range 0 to 155 months). At the time of data collection, 1931 (57.0%) patients were alive, and 1456 (43.0%) were deceased.

**Table 1:** Demographic and clinical characteristics of patients with brainstem gliomas.

Variables	Number	%
**Sex**		
Female	1535	45.3
Male	1852	54.7
**Age at diagnosis (years)**		
Mean±SD	26.86±23.244	
Median	18.00	
Range	0-103	
0-19	1161	34.3
≥20	2226	65.7
**Race**		
White	2715	80.2
Others	672	19.8
**PRCDA Region**		
Alaska	6	0.2
East	1187	35.0
North plains	258	7.6
Pacific coast	1740	51.4
Southwest	196	5.8
**Tumor Size**		
≤3cm	1259	37.2
>3cm	1134	33.5
Unknown	994	29.3
**Diagnostic confirmation**		
Positive microscopic confirm	2023	59.7
Others	1364	40.3
**Behavior code**		
Benign	113	3.3
Borderline malignancy	234	6.9
Malignant	3040	89.8
**Surgery**		
Yes	1479	43.7
None/Unknown	1908	56.3
**Radiation**		
Yes	1746	51.6
None/Unknown	1641	48.4
**Chemotherapy**		
Yes	1166	34.4
None/Unknown	2221	65.6
**Survival months**		
Mean±SD	44.22±44.404	
Median	24.00	
Range	0-155	
**Vital status**		
Alive	1931	57.0
Dead	1456	43.0

### Survival Analysis

**Figure 4:**
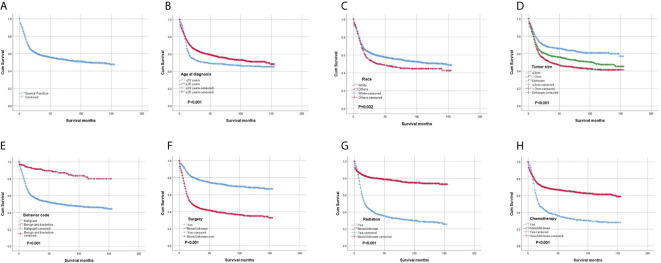
Kaplan-Meier survival analysis: **(A)** The overall survival for the whole cohort. The survival analysis of patients classified based on **(B)** age at diagnosis, **(C)** race, **(D)** tumor size, **(E)** behavior, **(F)** surgery, **(G)** radiation and **(H)** chemotherapy.

The OS rates at 1, 3, 5 and 10 years after diagnosis were 70.8%, 56.3%, 53.3% and 48.8%, respectively. A Kaplan-Meier curve was created to show the OS for the full cohort (**Figure 4A**). The Kaplan-Meier log-rank test indicated that the variables age at diagnosis (**Figure 4B**), race (**Figure 4C**), tumor size (**Figure 4D**), behavior (**Figure 4E**), surgery (**Figure 4F**), radiation (**Figure 4G**) and chemotherapy (**Figure 4H**) were possibly related to OS. The results of multivariate Cox proportional hazard regression analysis showed that race, age and sex were not independent prognostic factors. Patients with tumors less than 3 cm in size or benign or borderline tumors had a better prognosis. The results also showed that surgery was effective for improving prognosis, but radiation and chemotherapy did not help patients obtain a better prognosis. The results generated by the log-rank test and univariate and multivariate Cox proportional hazard models are listed in **Table 2**.

**Table 2:** Results of the log-rank test and univariate and multivariate Cox regression analysis.

Variable	Log-Rank Test	Univariate Analysis		Multivariate Analysis	
	P value	HR (95%CI)	P value	HR (95%CI)	P value
**Sex**	0.270				
Female		Reference		Reference	
Male		0.944 (0.851-1.047)	0.275	0.998 (0.899-1.108)	0.973
**Age at diagnosis** (years)	<0.001				
≤19		Reference		Reference	
≥20		0.802 (0.721-.892)	<0.001	0.908 (0.812-1.016)	0.092
**Race**	0.002				
White		Reference		Reference	
Others/Unknown		1.210 (1.069-1.370)	0.003	1.003 (0.885-1.137)	0.959
**Tumor Size**	<0.001				
≤3cm		Reference		Reference	
>3cm		1.662 (1.462-1.889)	<0.001	1.240 (1.079-1.424)	0.002
Unknown		1.408 (1.227-1.616)	<0.001	1.159 (1.008-1.332)	0.038
**Behavior**	<0.001				
Malignant		Reference		Reference	
Benigan and Borderline		0.212 (0.155-0.290)	<0.001	0.507 (0.366-0.703)	<0.001
**Surgery**	<0.001				
None/Unknown		Reference		Reference	
Yes		0.337 (0.299-0.380)	<0.001	0.337 (0.298-0.381)	<0.001
**Radiation**	<0.001				
None/Unknown		Reference		Reference	
Yes		3.651 (3.235-4.121)	<0.001	2.832 (2.465-3.254)	<0.001
**Chemotherapy**	<0.001				
None/Unknown		Reference		Reference	
Yes		2.188 (1.972-2.428)	0.001	1.234 (1.100-1.384)	<0.001

## Discussion

Brainstem glioma is a primary glial tumor that arises within the brainstem and is believed to be a heterogeneous group of gliomas. Some authors divided brainstem gliomas into 2 categories. Twenty percent are considered to be focal low-grade lesions with good prognosis, and the remaining 80% of tumors arise in and occupy the majority of the pons and are diffuse in nature and associated with poor prognosis (8–10). We could not define whether the tumor originated from the midbrain, medulla, or pons, and we also could not define whether the tumor was focal or diffuse based on the data available in the SEER database. Some authors have conducted cohort studies with data on high-grade glioma (11, 12) or low-grade glioma (13) based on the SEER database. Brainstem glioma is a relative rare lesion, and previous reports have included limited numbers of cases. We believe that this limitation might prevent us from obtaining a better understanding of the general characteristics of the patients diagnosed with brainstem glioma. The SEER program represents approximately 35% of the US population, and the data were collected from most parts of the United States (5). To better understand brainstem glioma, we conducted this large-scale cohort study including 3387 cases. To the best of our knowledge, this is the largest brainstem glioma cohort to date. We believe our report can explain the epidemiology and survival of patients with brainstem gliomas in the United States to some extent.

First, we wanted to conduct an analysis of cancer-specific survival. However, because the brainstem glioma was not the first malignant tumor in some patients, cancer-specific survival was not applicable for approximately 10% of the cohort. Progression-free survival cannot be adopted because we cannot obtain data about progression from the SEER database. To include all of the data and make the analysis accurate, we chose to analyze OS. The World Health Organization (WHO) grade classification is widely used by neuro-oncology doctors, but half of the tumors were not clearly classified according to WHO grade. Therefore, we chose the behavior code according to ICD-O-3; in our cohort, more than 80% of the tumors were malignant, which is in agreement with a previous study (3).

According to the report from The Central Brain Tumor Registry of the United States (14), in children and adolescents, brainstem tumors account for 10.8% of all primary central nervous system tumors. Brainstem gliomas were also reported to account for up to 20% or more of primary brain tumors (15). In our cohort, the highest incidence was found in individuals between 5 and 9 years old, and the median age at diagnosis was 18 years old. However, we found that brainstem gliomas could be diagnosed at every age. Children and adolescents (0-19 years old) accounted for 34.3% of all patients, and more than half of the patients were adults. The predominance of white patients was also very significant in our study, which is consistent with the findings of other cohort studies of gliomas based on the SEER program (13, 16). Males and females are almost equally affected. We have also analyzed the pathological classification of the tumors in these patients, with the intention of providing information that could be useful when biopsy or surgery cannot be performed but a treatment needs to be selected. According to a previous report, diffuse intrinsic tumors account for approximately 80% of all brainstem gliomas. These tumors are generally high-grade anaplastic astrocytoma (WHO grade 3), glioblastoma multiforme (WHO grade 4), or occasionally well-differentiated diffuse astrocytoma (WHO grade 2) (17). However, in our cohort, we found that the most common pathology type was ependymoma/anaplastic ependymoma and pilocytic astrocytoma. Glioblastoma accounted for only 9% of all cases with positive histology. Because the anatomical location of the tumor precludes surgery, radiation is the standard therapy for patients with diffuse intrinsic tumors (17), and tumor resection or biopsy is not recommended for some patients. In our cohort, 59.7% of the cases were diagnosed with a positive microscopic method. There could be some bias in our research of the pathological characteristics because the data were not fully included. We believe further investigation should be conducted to clarify the findings, and further research about molecular pathology is also needed.

In our study, we found that race, age and sex were not independent prognostic factors. However, patients with tumors less than 3 cm in size had a better prognosis than patients with tumors larger than 3 cm. We believe that timely diagnosis and treatment are essential for patients. Focal radiation therapy is the current standard of care for children with diffuse intrinsic pontine glioma (18). We also found that radiation therapy was chosen more frequently than surgery and chemotherapy. However, the results of the survival analysis showed that radiation and chemotherapy did not improve overall survival. The systematic review conducted by Xu et al. (19) also could not make definitive conclusions regarding whether radiotherapy can help patients obtain better survival. We believe that further high-quality studies are needed to establish the role of radiotherapy in the management of brainstem gliomas. Brainstem glioma is believed to be composed of a heterogeneous group of gliomas, and individualized treatment is needed based on pathology. Our results also showed that surgery can improve prognosis. The extent of surgery in some cases was difficult to clarify based on the code provided. In particular, biopsy and ventricular peritoneal shunts are widely used for patients with brainstem gliomas. We did not analyze the impact of surgery extent on survival. Based on the results, the prognosis of brainstem glioma is poor, and the median survival time is 24 months. Different attempts have been made to treat patients with brainstem gliomas (4, 18, 20), and we hope that more effective treatments can be discovered in the future.

There are several limitations of our analysis that must be considered. The availability of some important information was limited, such as information on the tumor location (midbrain, pons, and medulla), focality of diffuse glioma, molecular pathology, more specific treatment, tumor progression and so on. The specific location and molecular features are very important factors affecting progression, and molecular pathological investigations have been widely implemented in clinical practice. We hope that we can obtain more detailed information from the SEER database in the future. Although it would necessitate the inclusion of a substantial amount of information, it would assist in the realization of a better understanding of primary CNS tumors.

## Conclusion

Brainstem gliomas can be diagnosed at every age. Ependymoma/anaplastic ependymoma is the most common pathological diagnosis. The predominance of white patients was significant. The prognosis was poor, and the median survival time was 24 months. Timely diagnosis and surgery are effective in improving the prognosis, and individualized treatment is essential for patients. We suggest that more attention should be paid to the treatment of patients with brainstem gliomas.

## Data Availability Statement

The original contributions presented in the study are included in the article/supplementary material. Further inquiries can be directed to the corresponding authors.

## Author Contributions

YW and GZ: Conceptualization, methodology, and reviewing and editing. YW: Data curation, software, and validation. HL, XQ, LZ, and YW: Original draft preparation, and reviewing and editing. All authors contributed to the article and approved the submitted version.

## Conflict of Interest

The authors declare that the research was conducted in the absence of any commercial or financial relationships that could be construed as a potential conflict of interest.
